# Leisure and meaning in life

**DOI:** 10.3389/fpsyg.2023.1074649

**Published:** 2023-02-28

**Authors:** Seppo E. Iso-Ahola, Roy F. Baumeister

**Affiliations:** ^1^Department of Kinesiology, University of Maryland, College Park, MD, United States; ^2^School of Psychology, University of Queensland, Brisbane, QLD, Australia

**Keywords:** leisure, meaning, well-being, health, activity involvement, boredom, intrinsic motivation, serious leisure

## Abstract

How people engage in leisure is an important but frequently underappreciated aspect of meaning in life. Leisure activities range from highly engaging and meaningful to subjectively trivial. Leisure itself is largely defined by meaning: The essence of leisure lies less in the specific activity than in the subjective perception of freedom, choice, and intrinsic motivation. People desire their lives to be meaningful, and leisure activities offer varying degrees of satisfying the basic needs for meaning (here covered as purpose, value, efficacy, and self-worth). Leisure activities vary along multiple conceptual dimensions, such as active vs. passive, seeking vs. escaping, solitary vs. interpersonal, and we consider the implications of these for meaningfulness. The most common leisure activity in modern society, watching television, encapsulates some of the paradoxes of leisure and meaningfulness. The study of how leisure enhances meaning in life is rich and ripe for future research.

## Introduction

Research has abundantly confirmed the importance of both interpersonal relationships and work to the meaning of many lives (Bellah et al., 1985; Stillman et al., 2009; Lambert et al., 2010). More precisely, meaning is often found in interpersonal relationships, especially strong and close ones, as well as in meaningful work. However, the contribution of work to life’s meaningfulness is highly variable. Some people find it highly engrossing, fascinating, and rewarding, while others see it as little more than a tedious activity necessary to provide money to support life. Indeed, a surprisingly large category of people describes their work as “bullshit jobs” and think society would be perfectly well off if their job did not even exist (Graeber, 2013, 2018). For such individuals, family, romance, and other forms of social contact loom as the primary source of meaning.

In this article, we seek to examine another possible source of meaning in life: leisure. While for most people, leisure remains secondary to work in terms of priority, it can nevertheless infuse substantial amounts of meaning into life, along with boosting happiness and satisfaction. We seek to explain just how leisure contributes to satisfying people’s needs for meaningfulness. For example, meaningfulness often emerges from close relationship bonds, and leisure is an avenue for doing activities with friends and family (e.g., Crandall, 1979; Iso-Ahola, 1980, 1999). However, it is important to add that leisure activities are also done alone or in formal social contexts with weak interpersonal relationships, such as acquintances in structured programs.

When work is unsatisfying, leisure can potentially help fill the gap in meaningfulness, but it can also be meaningful in its own right. Although we focus on leisure’s potential contribution to meaning in life, we do not want to imply that work and interpersonal relationships, on their own without leisure, would not play an important role in people’s search for meaning in life. They certainly are important contributors, but we wish to highlight how and why leisure can make its own contribution. This is important because scholars, especially psychologists, have paid very little attention to the relationship between leisure and meaning in life. In contrast, some sociologists (e.g., Coalter, 1999, p. 513) have acknowledged psychological aspects and centrality of leisure choices and their “situated meaning.”

Examination of the relationship between leisure and meaning in life is important not only for its own sake but also, for better understanding the complexity and breadth of meaning in life in general. Furthermore, the examination is important now in the aftermath of the wide-spread effects of the COVID-19 pandemic (e.g., remote work). Much has been said about the so-called Great Resignation or “quiet quitting” from jobs. Could leisure replace work or provide opportunities for doing something one always wanted to do and thereby help make his/her life more meaningful?

Along with the great resignation, there is a trend of adopting shorter workweeks without pay cuts. In Iceland, 86% of workers are expected to adopt a 4-day workweek based on the experimental findings, and Belgium announced that employees are allowed to request compressing their work hours into 4 days. In the U.S. Congress, a bill has been introduced to reduce all standard workweeks to 32 hours. All of this has a potential to increase leisure’s importance to meaning in life.

Recent years have seen researchers shift away from a focus on the meaning *of* life to meaning *in* life (e.g., Steger et al., 2006; Steger, 2009; George and Park, 2016; Martela, 2020). The difference is in the amount of integration required: A meaning of life presumably integrates much of the life, including most or all of the important parts, whereas meaning in life can be limited to one domain and can comfortably ignore large and important aspects of the life’s meaning. Finding meaning in life is a less grandiose aspiration than determining the meaning of a life or indeed of all life. This shift increases the possibilities for recognizing and studying the contribution of leisure pursuits. Although leisure may not rise to the level of providing the meaning of life for most people, it can provide substantial increases in the amount of meaning *in* a life. Our focus is to advance understanding of leisure’s potential to enhance meaning in life. Nevertheless, we stipulate that leisure can enhance the meaning of life also. For example, millions of people around the world spend their leisure in volunteer work helping the poor, serving the church, or improving the environment, and that provides not only meaning in but meaning of life for them.

Another important point is that leisure does not have to make unique contributions to meaning in life. Kelly and Kelly (1994) provided evidence that the meanings people find in leisure often overlap with what they find elsewhere (e.g., in work and family). Nevertheless, leisure can still contribute important and substantial amounts of meaningfulness.

The paper will proceed as follows. First, we consider the defining characteristics of leisure. Next, we review evidence relevant to the question of how leisure can satisfy people’s various needs for meaning. Following this generally positive appraisal of how leisure contributes meaning, we review evidence concerning several problematic aspects of the meanings in leisure. Television watching, in particular, is often rated as low in meaningfulness and happiness, yet it has often been found to be the most frequently reported form of leisure in modern life. We then cover evidence about several key dichotomies in leisure, including solitary versus interpersonal leisure, casual versus ‘serious’ leisure, and whether leisure is primarily an escape from something else or is sought and valued in its own right. We then conclude with some broad observations.

## What is leisure?

Leisure can be defined by what it is and what it is not. First, leisure can be most easily defined by what it is not: It is not work, nor does it include other activities required for survival. Instead, leisure has traditionally been defined in three ways: As an activity or as time left over after work or as a subjective preception and experience (Iso-Ahola, 1980). Most agree that it cannot be defined as an activity because any activity could sometimes be defined as leisure. It does not make sense to define leisure in terms of time left after work either, because time does not tell us anything about meaning, antecedents and consequences of behaviors done after work. If a person spends many hours watching TV but does not like it, it is a poor leisure experience (Kubey and Csikszentmihalyi, 2002). There are numerous behaviors (e.g., chores, child care) undertaken after work that can be characterized as nonwork or free time, but few people would define them as leisure. There is a difference between the mere nonworking hours and extraction of meaning from these hours.

Multiple studies (e.g., Iso-Ahola, 1979, 1980; Shaw, 1985; Mannell et al., 1988) have shown that leisure is a psychological entity overwhelmingly defined by people’s perceptions of freedom. In other words, a sense of freedom more than anything else defines what leisure is to people. Importantly, leisure means freedom to choose to do or not to do something. Otherwise a sense of obligation arises and a sense of leisure is lost. Thus, the etymological roots of the word “leisure” are linked to the concept of freedom. Freedom to choose allows people to pursue their values, goals, and identities (Schwartz, 2004). Even though perceived freedom is a necessary condition for leisure, it does not guarantee a high-quality experience. One can freely choose to go and watch a basketball game, but if his/her team loses, this leisure experience would predictably be rated poor (Madrigal, 2003). It should also be noted that not all scholars, especially sociologists (e.g., Rojek, 2010), agree with our social psychological approach to defining leisure.

Nevertheless, research suggests that an opportunity for freedom and choice is valuable in and of itself because a choice provides the means for exercising control over one’s environment, thereby suggesting that the need for freedom and choice is biologically based. To this extent, Leotti and Delgado (2011) showed that the mere anticipation of personal involvement in an activity through freedom of choice recruited affective and motivational brain circuitry, specifically corticostriatal circuitry known to be linked to reward processing. Research has further shown the fundamental importance of freedom, in that individuals prefer freedom to choose even when it impairs their social welfare and can lead to tragic medical decisions (Botti and Iyengar, 2006; Botti et al., 2009).

The second most important characteristic behind the concept of leisure is intrinsic motivation, followed by “work-relation” and “goal-orientation.” In other words, it is freedom rather than lack of it, intrinsic rather than extrinsic motivation, final goals rather than instrumental goals, and low work-relation rather than high work-relation which increased people’s perceptions of leisure (Iso-Ahola, 1979). While the effects of the latter two were statistically significant, their effects on the perceptions were negligible compared to the former two, especially a sense of freedom. What this means is that any so-called leisure activity can turn into work-like activity when it is forced or has no sense of freedom associated with it.

In their classic study, Csikszentmihalyi and Graef (1979) showed that even such a supposedly pleasant leisure activity as being in a restaurant can be turned into anything but leisure when people are required to be there. All of this is also consistent with the research on the “overjustification” phenomenon which has shown that initially intrinsically motivating activities become work-like when they are externally sanctioned or extrinsically motivated (Deci, 1971; Lepper et al., 1973; Deci et al., 1999). According to Csikszentmihalyi and Graef’s (1980) data, sports and games are activities in which people feel most free.

In short, leisure cannot be defined as an “activity,” such that some activities are leisure activities while others are not, because almost any activity can be experienced as either leisure or work, depending on perceptions of freedom. The definition of leisure therefore resides more in the person’s attitudes toward and feelings about the activity, rather than in the activity itself. Freedom and intrinsic motivation contribute to the understanding of leisure as something that the person wishes to do and feels free to decide whether to do it or not.

It should also be noted that not only an opportunity to choose to do something in free time makes that chosen activity leisure but also a choice not to do something is an expression of freedom (Iso-Ahola, 2013). Thus, freedom gives people a license not to exercise!

It is easier to provide examples than a definition. Thus, leisure activities include hobbies, rest, entertainments, games, and sports (both as participants and spectators). Travel is also a popular leisure activity. Consumption of food, alcohol, and drugs is also a popular form. For all of these and others, the feeling of being free to do it or not do it is what makes it leisure. Travel can be required by work, and taking drugs may be required by addiction, and so forth, in which case they lose the character of leisure.

Regardless, the essence of leisure resides in its subjective meaning, as was well demonstrated empirically by Tonietto et al. (2021). Their findings indicated that perceiving leisure as wasteful correlated with lower happiness and greater depression, anxiety, and stress. Furthermore, priming the belief that leisure is wasteful reduced enjoyment of leisure. Unfortunately, in the achievement-emphazing society like the U.S., priming productivity at the expense of leisure is common, and the utility of leisure is seen as the relief it affords from costly cognitive control in labor-leisure relationships and tradeoffs (Kool and Botvinik, 2014).

Yet, research has shown the benefits of mentally disengaging from work during off-time. Individuals who are able to detach themselves from work during their off-job hours report higher life satisfaction and well-being and fewer symptoms of psychological strain (Sonnentag, 2012). Moreover, there appears to be a curvilinear relationship between attachment from work during nonwork time and task performance such that both high and low levels of detachment were associated with poor task performance (Fritz et al., 2010). This would seem to suggest that both too much and too little psychological separation between work and leisure is not good for employees’ well-being and job performance.

## Leisure and needs for meaning

We turn now to the core question of how leisure activities can contribute to meaning in life (if not of life). The underlying premise is that people are broadly motivated to find or instill meaning in their lives (Steger, 2009; Park, 2010). We enquire, therefore, how leisure pastimes may help accomplish this.

Meaning in life can be characterized as among those desirable things that many people want but are not sure quite what it is. Frankl’s (1976/1959) pioneering work on meaningfulness emphasized purpose as a fundamental and central form of meaning. Seeking to elaborate the notion of meaning motivation, Baumeister (1991a) proposed four somewhat distinct needs for meaning: Purpose, value, efficacy, and self-worth. Hence one way to elucidate the contribution of leisure to meaning is to analyze how various leisure activities address and potentially satisfy these four needs.

The four needs for meaning are a heuristic scheme. George and Park (2016) suggested purpose, mattering, and coherence, which initially seem different but on closer inspection are quite similar. (For example, value might seem to be missing from George and Park’s scheme, but they stipulate that the purposes must have value, and moreover, value is relevant to mattering.) A more thorough examination of how the different lists of meaning needs are actually quite similar can be found in Baumeister (2023).

### Purpose

Purpose means that the present activities draw meaning from the future, such as aspirational goals or fulfillment states. Leisure activities vary widely as to how purposive they are. The single most common leisure pastime in modern Western civilization is watching television, which typically is lacking in either goals or fulfillment states. (That may explain why television watching is rated as among the least satisfying or pleasant of daily activities; Kubey and Csikszentmihalyi, 2002). At the opposite extreme, creative hobbies such as playing a musical instrument or painting have abundant goals and sometimes offer fulfillment states (i.e., the ecstasy of artistic creation).

Goals in leisure pursuits can be short-term, long-term, or both. As examples of short-term goals, sports, games, and athletic pursuits often come with proximal goals, such as winning the game, finishing the climb or hike, or solving the puzzle. Nevertheless, these activities are psychologically meaningful because there is often a strong relationship between challenge and enjoyment in them. Abuhamdeh and Csikzentmihalyi’s (2012) data showed that the challenge-enjoyment relationship is strongest for intrinsically motivated, goal-directed activities. The authors suggested that the motivational context (intrinsic-extrinsic motivation) and the nature of the activities (goal-directed or not) have to be considered to understand optimal challenges in sports and games and other leisure activities.

To be sure, leisure can involve long-term goals as well. So-called serious leisure (see later section) often is serious precisely because of commitment to long-term goals, such as playing in a local band or volunteering to help the environment. Moreover, even when the goals in leisure pursuits may be short-term, people repeat their leisure activities. For example, they do not just play tennis one time but rather tend to play frequently or even regularly. If done with family and good friends, leisure activities can become meaningful additions to people’s lives. Thus, they do not act as if the activity has enabled them to reach a goal — they may select the same sort of goal the next time they play. Short-term goals may not contribute much to the meaningfulness of a life as a whole, but they can add plenty of meaning into the life along the way. (They may try to win each tennis game; after all, scoring and winning are inherent to the game.) In contrast, some leisure activities do involve longer-term purposes. For example, singing in a choir or volunteering for community service may be undertaken month after month, year after year. The participation can be cumulative, such as enabling the choir to flourish or helping a series of individuals to have a better life.

### Value

Philosophical and sociological conceptions of value can be quite complex. Heinich (2020) has observed, among other insights, that it is best to focus on how individuals bestow or judge value, rather than treating value as an inherent property of things. Assignment of value combines properties of the object, characteristics of the person making the judgment, and the situational context. She resists reducing value to morals or to normative guides on how to act. Ultimately, she says, value is neither objective, nor subjective, nor arbitrary. In contrast, consumer psychologists tend to start by equating value with the monetary price of an item, but a more in-depth analysis of what consumers value led Almquist et al. (2016) to delineate 30 different elements of value, which can be sorted roughly into four master categories: Functional value, emotional value, life changing value, and social impact.

These apply to leisure in different ways. Leisure is not generally functional, although the leisure enthusiast may pay close attention to which products and accessories are most functional. (As example, note the ongoing refinements in skis over the past half century, which have made skiing much easier and more pleasant.) Emotional value is presumably the most frequent reason that people choose particular forms of leisure, including even the wish for vicarious emotional experience from watching television. Life change may occur, such as if dabbling in guitar to relax after work gradually moves into cultivation of musical talent and public performance. Last, some people may choose forms of leisure that have positive social impact, such as helping the homeless or volunteering at a recycling center.

Whereas Almquist et al. (2016) sought to cover the operation of values in consumer purchases, and Heinich (2020) undertook to analyze all forms of value, our emphasis is on how leisure contributes to the value aspect of meaning in life. The need for value is a matter of finding a way to regard oneself and one’s life as good. Some leisure pursuits enable one to claim value based on belonging to a socially admired category of persons (e.g., musician, painter, sailor, athlete), while others contribute to the betterment of society (e.g., volunteer work). Or, to put it another way, all theorists and measures include purpose as vital to meaning in life – but purposes are not all equal, and most people seek purposes that have positive value.

The origins of the concept of leisure (including the etymology of the word) involve being freed from the duties, obligations, and other necessities of life. This reflects a simple view of life as divided into things one must do in order to survive and things that one wishes to do. Self-determination theory (Deci and Ryan, 1985) began with research on the difference between intrinsic and extrinsic motivation. Working to make a living is essentially extrinsic because it is driven by external demands. In contrast, leisure is assumed to be largely intrinsically motivated. Freed from the necessity to comply with external demands, one is able to do what one wishes to do. The essence of intrinsic motivation is that one performs the activity for its own sake, such as for the inherent pleasure of doing it, rather than to achieve external goals. Sailing provides a useful example. At some times and places, sailing was a crucial way to travel. One sailed in order to reach a destination so as to pursue one’s business there. It was a means to an end. In contrast, the modern recreational sailor often has no destination in mind and sails for the pure joy of the activity. Typically, one sails from one’s dock or harbor out into open water, cruises around for a while, and then returns to the starting point.

The value of work as a centerpiece of life has eroded among an increasingly greater number of people. Some people are willing to forfeit or forgo a substantial amount of their salary for more free time, and half of all American workers would choose a different type of work if they had to do it all over again (Marin and Gegax, 1997). At the same time, research has shown that involvement in meaningful nonwork activities helps people to detach from paid work, which in turn is associated with greater well-being (Sonnentag, 2012). When combining all of this with research showing that people overwhelmingly prefer experiences over possessions (Van Boven and Gilovich, 2003; Carter and Gilovich, 2012), it becomes clear that people are yearning for more meaning to their lives through means other than work, most notably through leisure that enables them to do personally meaningful activities.

However, it is not a question of substituting leisure for work but rather, providing an additional source for meaning in life. The problem is that 51% of the U.S. employees, according to a 2014 Gallup poll, do not feel involved in, enthusiastic about and committed to their work or workplace (and 17.5% “actively disengaged”) and would rather do something else if they could (Adkins, 2015). But because most people cannot switch jobs, they are stuck and in a way, forced to turn to other sources of meaning in life, such as activities done with friends and family. It, then, is not surprising that people value experiences much more highly than material possessions (Van Boven and Gilovich, 2003; Hunnicutt, 2020). A recent study found that valuing one’s experiences is positively correlated with perceptions of meaning even after controlling for purpose, mattering and coherence (Kim et al., 2022).

Thus, the contribution of leisure activities to value in life is complex and multifaceted. Some pastimes have a strong moral component, such as volunteer work. By working to help people less fortunate than oneself, or to clean up the environment, or to save animals, or to help one’s church, people can add value to their lives. In contrast, watching television, indulging in alcohol or drugs, or prostitution would seemingly add little value. Indeed, such leisure pastimes are regarded by some as destructive and unhealthy activities that detract from the total value for individuals and society. It must be acknowledged, though, that even these seemingly unhealthy activities have some remeeding value, in that they can positively contribute to mental health and positive emotion in the short term.

### Efficacy

Efficacy refers to the sense that one is making a difference, that one’s actions accomplish something. This is absent from some leisure activities, but is central to others, such as the examples of tennis and sailing. Thus, activities vary in how conducive they are in facilitating a sense of accomplishment, a sense of using one’s abilities to accomplish something personally and interpersonally meaningful and worthwhile. Learning to play a musical instrument or a skill-based sport requires practice so as gradually to build up one’s abilities. Successfully playing a complex piece on the piano or skiing down a steep hill seems likely to furnish a sense of efficacy, even if they fail to have any discernible or lasting effect on the external world. Likewise, winning a chess or card game furnishes a sense of efficacy, even if there are no lasting consequences (either for self or society).

At the same time, the complexity of leisure is apparent. Even those activities that do not seem to be ‘wholesome’ can be efficacious. For example, listening to music can improve one’s mood and thereby mental health in the short term. Similarly, moderate or social drinking can facilitate or “lubricate” meaningful social interactions (Crandall et al., 1980). Thus, it is more constructive to look at these types of activities in terms of their harmfulness than making moral judgments about them. Almost any leisure activity can be harmful when taken to the extreme (e.g., marathon running). Again, the essence of leisure does not lie in the activity but rather, in its subjective meaning.

It can be considered remarkable how many leisure activities embody the cultivation of efficacy for tasks and skills that have no pragmatic utility in normal life. Many sports rely on highly specialized muscular skills that bear no resemblance to any earnest activity. Unlike swimming and jogging, which have at times some practical utility in being able to move about in water or on land, tennis and basketball rely on cultivating fine motor skills that are useless for anything else. Nevertheless, the satisfaction of achieving efficacy at these activities can presumably add meaning to life. It has been found that “serious leisure” (i.e., time spent above an individual’s average) was positively related to work-related self-efficacy (Kelly et al., 2020). This presents intriguing possibilities for future research: Does leisure contribute significantly to meaning in life on its own or does leisure enhance work performance and self-efficacy and thereby increase meaning in life (a mediation effect)?

### Self-worth

The fourth need for meaning involves finding some way to view oneself as a person of worth. This typically derives from comparison to others: By pursuing valued goals in an efficacious manner, one achieves self-worth. In practice, Baumeister (1991a) observed that this often takes the form of feeling superior to others. In any case, some leisure sports offer opportunities to feel good about oneself. Again, the morally virtuous leisure activities furnish a sense of being a good person (both in one’s own mind and sometimes in other people’s estimation). Likewise, the amateur athlete or artist can enjoy successes along with admiration of others.

To be sure, in principle the boost to self-worth does not have to rely on social comparison. Merely performing a leisure activity for its own sake could increase a sense of self-worth, especially if the activities are based on using one’s skills (Csikszentmihalyi et al., 1977; Sheldon et al., 1996; Reis et al., 2000). Furthermore, an Australian study showed that those unemployed individuals who engaged in challenging activities, both social (e.g., sport and dancing) and solitary (e.g., hobbies, reading), reported higher levels of self-esteem than those unemployed whose leisure was dominated by “doing nothing” and watching TV (Winefield et al., 1993). This clearly demonstrates the potential of leisure activities adding meaning to people’s lives and for maintaining their self-worth, even for unemployed individuals. In general, self-esteem is linked to actual and anticipated evaluations of self by others (e.g., Leary and Baumeister, 2000).

In Veblen’s classic (Veblen, 1953/1899) analysis of the leisure class from the gilded age (late 19th century), self-worth was presumably a central motivation among the people he observed. The purpose of conspicuous consumption was to garner the admiration and perhaps envy of others. Notably, conspicuous consumption does not establish worth via virtuous deeds, successful achievements, or skillful performances. Rather, it showcases one’s wealth, presumably invoking the assumption that rich people are the elite of society. If nothing else, one envies them for their wealth, and being envied may contribute to a sense of superiority. However, engagement in leisure is not just a matter of flaunting one’s social status, or only a matter of intrinsic reasons. At times and in certain situations, both intrinsic and extrinsic motivators are present in leisure pursuits (Mannell and Bradley, 1986; Mannell et al., 1988).

## Problematic aspects of seeking meaningfulness in leisure

Thus far we have argued that various forms of leisure can satisfy the needs for meaning — some far more than others. Having elucidated the positive case for leisure’ contribution to meaningfulness, we turn now to some of the problems that may reduce such contribution.

As stated earlier, freedom is the essential, defining feature of leisure. Freedom is highly desired, and in general people express a pervasive and sometimes strong wish for greater freedom (Iso-Ahola, 1980; Mannell et al., 1988). Yet when they get freedom, they often seem not to know what to do with it (Csikszentmihalyi, 1999), resulting in boredom in leisure among other things (Iso-Ahola and Weissinger, 1987, 1990). This suggests that people often find extrinsically motivated activities, including work, to be burdensome, even aversive, but they wish to not have to do what others tell them to do. When they obtain freedom, however, they may find themselves at a loss as to what to do instead of extrinsically mandated activities. In fact, Mannell and Bradley’s (1986) experiment showed that individuals who believe that they have less control in their lives find free time threatening and therefore achieve high quality experiences in more structured and restricted settings. Consistent with this, it has been found that perception of having too much leisure time correlates with lower subjective well-being (Sharif et al., 2021). The fact that an average American spends 3–5 h per day watching TV, depending on demographic groups studied (Grontved and Hu, 2011; ATUS, 2018), may in part reflect a psychological threat that leisure poses to many because of not knowing what to do with unstructured free time. This and other paradoxes are inherent in television watching. We begin this section with a consideration of this popular leisure activity.

### Television watching, leisure, and meaning in life

Among modern citizens in western civilization, watching television stands out as the most frequent leisure activity and indeed one of the main ways that people spend their time (mainly after work and sleep). According to the American time use survey (ATUS, 2018), an average American spends 55% if his/her leisure time watching TV, with the number of hours varying from 2 h 46 min to even 8 h depending on the groups of individuals studied. In general, the older, less educated and less affluent people watch more TV. Research has shown that such a prolonged TV watching is associated with an increased risk of type 2 diabetes, cardiovascular disease, and all-cause mortality (Grontved and Hu, 2011). It should be added that it is not just TV watching but spending an inordinate amount of time daily peering at smartphones (5 h on average) that makes people passive participants in leisure. Although we focus on TV watching in this analysis, it should not be forgotten that the total “screen time” is much greater than the mere hours of TV watching. Thus, TV watching understates the time spent in passive activity.

The high amount of television watching would be readily understandable if television watching were the most pleasant and satisfying of activities. But it is not. If anything, people report surprisingly low happiness, satisfaction, and meaningfulness associated with watching television. Using the experience sampling method, Kubey and Csikszentmihalyi (2002) found that heavy viewers (more than 4 h per day) enjoyed TV watching less than light viewers (less than 2 h/day), with the authors suggesting that twinges of unease and guilt in part depreciate the enjoyment. The heavy viewers also felt more anxious and less happy (than the light viewers) in unstructured situations, such as doing nothing, daydreaming or waiting in line. Something other than the quest for deeply rewarding leisure activities must explain the high amount of viewing.

Before dismissing television as a futile, self-defeating exercise of misguided quest for satisfaction, however, it is necessary to acknowledge that television audiences may derive some meaning from watching, even if it is not immediately obvious. Intellectuals and even ordinary people may not notice the meaningfulness of television watching or may be reluctant to admit it, possibly based on a stigmatizing stereotype that watching television is an unproductive activity. Television watching does furnish people with a sense of freedom, with the “felt freedom” in TV watching being only second to sports and games, and third after sports/games and reading in “I wanted to do it” (Csikszentmihalyi and Graef, 1979, 1980).

Another potential benefit of watching television is social connection. As already noted, people rate connecting socially with other people as a major source of meaningfulness (e.g., Lambert et al., 2010). Gabriel, et al. (2020) showed that people often feel a strong sense of connection with others while watching television, especially when watching in the presence of others. This included even strangers, that is, people felt a social bond with others who were watching the same event. This fact may contribute to the often-remarked finding that people prefer to watch sports events live rather than after a delay (e.g., Vosgerau et al., 2006) — presumably because when watching the show live, they know that many others are also watching exactly the same contest and having similar reactions. In connection with this, some commentators have suggested that the proliferation of television choices has actually contributed to the fragmentation of modern society. Murray’s (2012) book Coming Apart, which analyzed the disintegration of social cohesion in modern America, began its analysis with the night before the assassination of president Kennedy — when roughly a third of Americans were all watching the same show (*The Beverly Hillbillies*). Modern on-demand streaming services make it much easier for a viewer to choose to watch a favorite show at any time that is convenient, but perhaps in the process some connection to others in society is lost.

There may well be a second, occasionally even more important contribution to meaning by television watching (Gabriel et al., 2016). Watching may immerse the viewer in what Gabriel et al. dub “surrogate social worlds.” Favorite television shows involve regular viewers in the fictional web of social relationships among the main characters. Although these relationships are not real, viewers may lose sight of that fact. Gabriel et al. point out that the human brain is not adapted to make strong distinctions between what is real and what is imaginary. People watch these favorite shows especially when they are feeling lonely, which is one indication that watching can provide a sense of belongingness. Experimental studies have confirmed that reflecting on threats to close relationships led to feelings of rejection, bad moods, and temporary loss of self-esteem – but reflecting on one’s favorite television shows eliminated those effects (Derrick et al., 2008).

In some cases, people develop what Gabriel et al. (2016) label “parasocial relationships,” the feeling that one has a personal connection either to a character on a fictional show or the actor or actress who portrays that character. Such a one-sided interpersonal bond presumably provides a sense of meaning despite the apparent futility of having a relationship with someone who does not know you exist.

Ease and convenience may well contribute to the high rate of television watching despite its frequently meager returns on meaningfulness. Most modern citizens have access to television. Watching it requires relatively little in the way of active decision or effort. Iso-Ahola (2015) has noted that work and other demanding activities can induce a state of ego depletion, that is, temporarily reduced willpower emanating from work results in a decrease in self-control and executive function (e.g., Baumeister and Vohs, 2016). For a depleted individual, watching television may appeal because it makes relatively few demands. Motivating oneself to engage in strenuous sport or musical practice may seem extra difficult to them, whereas turning on the television is quite easy. People may often say (Kaplan and Berman, 2010) that they believe they should not watch so much television and should engage in productive or constructive activities instead, but in a depleted state, their self-control to live up to those goals is reduced, and perhaps the appeal of a pastime that makes no executive demands on the self is extra salient.

Indeed, a growing body of research suggests that “people are unable to resist spending more time engaging in this activity (TV watching) than they would consider healthy or desirable” (Kaplan and Berman, 2010, p. 49). As Kubey and Csikszentmihalyi (2002) reported, for many TV watching borders being an addiction, in the words of one respondent: “If television is on, I just cannot take my eyes off it, I do not want to watch as much as I do, but I cannot help it, I feel hypnotized when I watch television.” Viewing begets more viewing, as the authors suggested. Finally, Csikszentmihalyi et al. (1977) reported that such important indicators of psychological well-being as mental alertness, sense of control, sense of competence, and sense of challenge were at their lowest when watching TV, while these indicators were at their highest when playing sports and games.

All of this evidence points out that the best leisure experiences are freely chosen activities in which people can use their skills and meet challenges. So, for example, recreational tennis and racquetball players do not choose to play against those who are much better or much worse but rather, those who are equal in skills or slightly better. Such opponents push one to the outer limits of his/her skills and provide a balance between challenges and skills, a prerequisite for “flow” experiences (Csikszentmihalyi, 1999). These experiences are based on “active” involvement rather than being passively absorbed in receiving information. Their range varies greatly from sports and games to travel and painting. They do not have to be physically demanding activities, but merely cognitively engaging like in reading interesting novels. Social interaction is a big part of leisure and may in part be so because it is a cognitively stimulating activity, an “active” activity.

### Active versus passive

An ironic paradox of leisure participation, however, is that while *active* activities like sports and games help satisfy the basic needs and provide rewarding and meaningful daily experiences, people spend most of their free time in *passive* activities like TV watching — even while describing them as the worst experiences (Kubey and Csikszentmihalyi, 2002). In contrast, a long line of research (Iso-Ahola, 1997) has shown that those who maintain an *active* leisure lifestyle and actively participate in specific activities have higher perceived physical, mental, and social health. Roberts et al. (1989) found that people with a “rich” leisure pattern (i.e., more varied and frequent involvement than the average for the sample) were the healthiest group, whereas those with “impoverished” leisure were least healthy of all participants in their study. Participants’ health status was a combination of four physical health indicators and two self-ratings of health. Other studies (London et al., 1977) have shown a significant positive relationship between leisure participation and indicators of mental health (i.e., reduced depression and anxiety). To be sure, this does not exclude reciprocal causality, in that healthy people are better able to engage in various leisure activities.

Nevertheless, the Roberts et al. study and other similar studies indirectly reveal leisure’s important contribution to meaning in life, namely, through close relationships and social interaction. To be sure, friendships can be established and meaningful social interactions had at work, but most of the time and for most people, meaningful social relationships take place in leisure time, be it family activities or doing something with good friends. Thus, it is not surprising that social relations/interaction and how time is spent correlate highest with happiness, with social interaction being fundamental and “necessary” for happiness (Baumeister and Leary, 1995; Diener and Seligman, 2002; Diener et al., 2018). Nor is it surprising that social connection is a major determinant of morbidity and mortality (Rook, 2015; Holt-Lunstad, 2021) and that social connection mediates the effect of positive affect on physical health, that is, as positive emotions increase so do positive social connections, resulting in better physical health (e.g., Kok et al., 2013).

## Social leisure

A critical dimension of leisure is that it is largely a social experience and phenomenon (e.g., Crandall, 1979; Crandall et al., 1980). Samdahl (1991) found that some type of social interaction occurred in 54.4% of the occasions labeled leisure, and much of this (44.7%) was characterized by informal social interaction. Many studies (e.g., Larson et al., 1986; Argyle, 1992) have reported that having friends and companions with whom to do enjoyable activities together is related to higher psychological well-being. Similarly, another study (Graef et al., 1983) found that “socializing” was as intrinsically motivating as other “active leisure” pursuits, and provided high levels of happiness. In order to achieve these benefits, however, it is important that people are able to regulate their social contacts and interactions; regulation of social interaction is “an optimizing process” (Altman, 1975) in which people have to be able to control when and with whom to socially interact, sometimes shutting themselves off from others and at other times opening up themselves for interpersonal contacts. This also means that being alone is not necessarily a negative thing—as long as people choose it. A recent study by Uziel and Schmidt-Barad (2022) supported these ideas by demonstrating that people rate themselves as unhappiest when they do not choose to be others but still end up in unwanted social situations. But occasional times being alone bring happiness as long as it is freely chosen.

Social interaction is both motivation for and benefit of leisure participation (Iso-Ahola, 1999). Sheer socializing with friends and companions becomes motivating and rewarding at the same time, whether it is escaping routine social contacts (i.e., work mates and family members) or seeking interpersonal rewards from doing things with best “buddies.” Copp (1975) reported that for hunters, being with friends was as important as getting away from the usual social contacts. This regulation enabled them to achieve an optimal and ideal level of desired social contact and interaction. It is then not surprising that Crandall (1979) concluded that the “best” leisure activities are those that involve activity *and* friends. Its is worth noting that the greatest amount of time with family members was spent in maintenance and passive activities (e.g., TV watching), while active pursuits were much more frequent with friends, with more positive experiences realized with friends rather than with family members (Larson et al., 1986). This, then, expresses the essence of leisure: doing what one wants to do in his or her free time and doing it with whom and when he or she wants to (Iso-Ahola, 1999). Unquestionably, such leisure adds a significant amount of meaning to one life.

## Seeking versus escaping

Besides social connection and social interaction, leisure contributes to meaning in life through psychological benefits derived from free-time engagement. Mannell and Reid (1993) studied 416 Canadian managers and professionals to determine how they organize work and leisure in their lives and psychological benefits they derive from both. Results revealed that group differences could be accounted for by two independent factors: the extent to which these managers used leisure rather than work to seek out personal and interpersonal rewards and satisfactions, and the extent to which they used leisure rather than work to escape personal and interpersonal environments. In other words, improved psychological well-being is attained when people use their leisure time to seek personal rewards (e.g., a sense of competence through sports and games) and simultaneously escape personal difficulties and problems, as well as to seek interpersonal rewards (e.g., friends’ company) and simultaneously escape the routine interpersonal world (e.g., workmates and family members).

As such, results supported a 2-vector theory of leisure motivation (Iso-Ahola, 1989, 2022), according to which people use leisure to seek personal rewards from engagement and to simultaneously escape personal problems and the routine environment (not just work) on one hand and to use leisure to seek interpersonal rewards by doing things with friends but simultaneously escaping or leaving the routine interpersonal world behind on the other. In other words, leisure engagement brings meaning to people’s lives, because it enables them to pursue personal rewards in skillful activities and interpersonal rewards in social interaction with friends, but also at the same time allows them to escape or leave behind every day personal issues and usual interpersonal contacts. Indeed, Baumeister (1991b) proposed that the modern self can be burdensome and stressful, and people have acquired a wide assortment of activities specifically designed to escape from self-awareness.

The fact that Mannell and Reid’s and others’ (e.g., Snepenger et al., 2006) data have strongly supported the theory suggests that through the two dimensions (seeking and escaping), participation in leisure activities adds significantly to people’s felt meaning in life. Thus, in leisure, individuals can pursue such intrinsic rewards as self-development and feelings of competence and interaction with friends, as well rewards from being able to leave behind the usual personal environment and perhaps forced interpersonal contacts (e.g., workmates). A recent study showed that freely chosen social interaction had the strongest positive correlation with subjective well-being, sense of meaning, and perceived control, but being with others not by choice had the strongest negative relationship with subjective well-being (Uziel and Schmidt-Barad, 2022). It is proposed that of the two dimensions, seeking rather than escaping is more conducive to meaning in life, but this remains to investigated empirically.

## Serious versus casual leisure

We have emphasized how diverse leisure pursuits are. One important dimension along which they vary is seriousness. Some leisure pursuits may be trivial and frivolous, such as playing a Sudoku game to pass the time, others may become quite serious, such as the amateur musician who spends hours practicing each day, joins an ensemble or local band, follows a long-term plan for skill improvement, and performs for paying audiences. The latter may still regard music as a hobby and rely on his “day job” for most of his income and to support his family. As another example, there are people who occasionally play a game of cards for fun — and others who play almost every day after work, systematically hone their skills, and seek out intense competition in national tournaments. This is particularly true of playing video games. Playing these games is an interesting case because of its increasing popularity among youth and because one can turn into a professional and earn lucrative living doing so. Undoubtedly, many, if not all, began playing these games for sheer intrinsic interest, but for some it grew into a serious leisure activity, and for the best, a profession on its own.

According to Stebbins (1992, p. 3), serious leisure is “the systematic pursuit of an amateur, hobbyist or volunteer activity that participants find so substantial and interesting that they launch on a career centered on acquiring and expressing its special skills, knowledge and experience.” It is the opposite of casual leisure (e.g., TV watching and eating) that is not classifiable as amateur, hobbyist or career volunteering. Although serious leisure can be anything from amateur astronomy and archeology to barbershop singing and highly committed community service (e.g., volunteer work at a food bank), what is common to all participants in these activities is strong identification with and deep meaning derived from their pursuits.

However, it should be noted that Stebbins’ idea of serious leisure has not been accepted without criticism. Veal (2016) has suggested that serious leisure and casual leisure are not binary categories, but instead, serious leisure should be viewed as a continuum. (We find this point persuasive; most psychological phenomena exist on continuums.) Accordingly, most leisure activities are participated in with varying degrees of seriousness.

Serious leisure is different from project-based leisure (Stebbins, 2005), which refers to one-time special leisure occasions, like attending festivals and graduations or preparing and attending birthdays and Christmas get-togethers (baking, decorating etc.). Although such leisure episodes can be rewarding and meaningful, their effects on meaning in life are likely to be short-term compared to serious leisure. However, for many parents and grandparents, life’s meaning is in seeing and experiencing their children’s and grandchildren’s growth and achievements. To them, leisure occasions (e.g., birthdays) become special and memorable, adding significantly to meaning in life even if they are not experienced as often and regularly as serious leisure pursuits (e.g., volunteering).

Serious leisure is similar to “recreation specialization” that has mainly been studied in outdoor contexts among boaters, hunters, fishermen, campers, and birdwatchers. Essentially, the person is highly devoted to a particular leisure activity. The recreation specialist engages repeatedly and regularly in that activity, as opposed to having different leisure activities. Obviously, devotees of serious leisure typically do the same. Specialized recreation may differ from serious leisure in that many participants in specialized recreation do not seek to cultivate advanced levels of skill. They may favor a particular hobby or activity and even feel personally invested in and committed to it (e.g., social bridge and dancing; Scott and Godbey, 1992, 1994; Brown, 2007), but they tend to eschew skill development and expertise in them. In other words, people participate in these activities regularly to derive enjoyment from being able to use their skills in freely chosen activities and good social company, while not striving to become highly skilled competitors and experts. As an example, one of the present authors has skied regularly for many years and gives priority to good skiing opportunities, but makes hardly any effort to improve his skills, being comfortable identifying himself as a permanently intermediate level skier.

Serious leisure can satisfy all four of the needs for meaning, and not just on a temporary basis but over a long period of time. Undoubtedly, it contributes considerably more meaning to life than casual leisure. Specifically, serious leisure pursuits involve purposive activity, with goals and anticipated fulfillments extending far into the future. It becomes a key source of value in the person’s life (and we assume it is typically consonant with the person’s other main values). Most serious leisure pursuits provide a sense of efficacy, whether from skillful performance or virtuous community service. Last, we suspect people are typically proud of their serious leisure activities, so that any drive toward self-worth gains some satisfaction that way.

Serious leisure typically enjoys several additional features of meaningfulness. To become serious, the leisure activities must resonate with the self, and expressing the self is one component of meaningfulness (see Baumeister et al., 2013). Thus, they are chosen carefully based on the self. This personal meaningfulness can even enable some serious leisure activities to replace work as the most meaningful aspect of life other than family and social relations, as Stebbins (1992) showed with amateur archeologists and astronomers. We speculate that the rise of the internet has increased these capabilities, because they make it easier for the amateur to connect with others who share that passion and make it possible to spend countless hours in such online activity. Playing video games attests to this point.

In many cases, the person forms social relationships with others who share the same activity, whether it be amateur astronomy or birdwatching, and relationships contribute meaning. Meaning is also crucial in integrating experience across time (Baumeister and Vohs, 2016). Serious leisure activities often require substantial commitment over long periods of time. Mere quantity of time is, of course, not sufficient to qualify an activity as serious leisure. To return to the obvious example: On average, people spend more time watching television than in any other activity, and few people regard television watching as either a serious leisure pursuit or a personally meaningful and satisfying activity. As another example, “hanging out” and associated drug use is common among youth.

Research has shown that involvement in serious leisure correlates positively with meaning in life, personal growth and improved health, enhanced social relationships, positive affect and life satisfaction, and work-related self-efficacy (e.g., Baldwin and Norris, 1999; Kim et al., 2011, 2015; Heo et al., 2013; Phillips and Fairley, 2014; Kelly et al., 2020). All this evidence suggests that leisure pursuits, especially serious leisure, can significantly add to meaning in life, if not meaning of life, and even compensate for barren work. The ideal situation, of course, would be that both work and leisure together (or separately) increase the meaning in and of life.

## Discussion and conclusions

The essence of leisure is not in the activity but rather in its subjective meaning. In particular, what makes something qualify as leisure is that it is experienced as free choice, where intrinsic motivation can be the deciding factor (unlike most work). In general, people express a high desire for freedom — yet when they get more free time, they often do not know what to do with it, so many leisure hours are dissipated in trivial and unsatisfying pursuits such as watching television. For many (though certainly not all) people, the desire for freedom may often be more a matter of wishing to be free of the external demands of work than to be able to engage in a particular activity. This tension is evident in one of the basic dimensions along which leisure pursuits vary, that is, escaping rather than seeking. Personal meaning is undoubtedly involved in both: One wishes to escape from work and other activities that are experienced as extrinsically motivated, or one seeks pleasure and sometimes meaning by engaging in activities that one regards as strongly intrinsically motivated. Escapist motivations for leisure are also suggested in the widespread prevalence of passive leisure activities, in contrast to the more active sorts of leisure. Yet the active ones are generally rated as more fulfilling than the passive ones.

Leisure has some power to add meaningfulness to life, but only if it is not seen as wasteful. Research has shown that those who believe that leisure is wasteful score lower in happiness and well-being and higher in depression, anxiety, and stress (Tonietto et al., 2021). Perceiving leisure as wasteful obviously indicates that leisure is not seen to contribute to life’s meaningfulness. Yet, leisure may be particularly appealing to those individuals for whom work (and perhaps family) fail to provide satisfactory levels of meaningfulness. In terms of Baumeister’s (1991a) four needs for meaning, leisure offers some opportunities to satisfy each of them. In leisure, purposes tend to be short term, such as skiing down the slope or winning the game, but some can engage longer-term and thus more meaningful goals. How values are reflected in leisure pursuits may be a promising topic for future research, but the role of value is evident in the greater valuation of experiences as compared to owning possessions (Van Boven and Gilovich, 2003). Moreover, volunteer work and other morally virtuous leisure activities seem highly likely to increase meaning. Many active leisure pursuits involve skills, the exercise of which undoubtedly furnishes a sense of efficacy. The volunteer work would likewise be an important basis for the sense that one’s leisure activities are making a positive difference in the world. Last, self-worth can be bolstered by leisure activities that enable competitive success, virtuous contribution to the betterment of society, and possibly other pathways.

Our analysis suggests multiple directions for future research. First steps would include directly testing hypotheses that participation in (some) leisure activities is linked to higher meaningfulness in life—and, importantly, demonstrating which leisure pursuits cause people to experience more meaningfulness, and why so. A related hypothesis would be that people who report higher search for meaning (as contrasted with the presence of meaning) may take up particular leisure pursuits in order to satisfy that unmet need for meaning. Additional hypotheses would be that meaningfulness is particularly gained by leisure pursuits that are long-term rather than short-term, interpersonal rather than solitary, active rather than passive, and seeking rather than escapist. The relationship between leisure and meaning in life also raises interesting theoretical questions. Assuming that meaning in life consists of components (mainly work, family/interrelationships, and leisure), Are the effects of these components additive or interactive? Or, are the effects compensatory? And what are their relative weights? How does leisure’s contribution compare to that of work and interrelationships – and how does this contribution vary as a function of situations and groups of individuals?

Although the time available for leisure has fluctuated widely throughout human history and prehistory (Hunnicutt, 2020), and across different cultures and walks of life, leisure appears to be here to stay as an important fixture of modern life. And whereas the available time for leisure has varied in both directions, the diversity of opportunities for leisure pursuits has expanded dramatically. How people choose to spend their leisure time is a highly variable but important form of self-expression — and, ultimately, a variable but sometimes important contribution to the meaningfulness in and of life.

## Author contributions

Both authors listed have made a substantial, direct, and intellectual contribution to the work and approved it for publication.

## Conflict of interest

The authors declare that the research was conducted in the absence of any commercial or financial relationships that could be construed as a potential conflict of interest.

## Publisher’s note

All claims expressed in this article are solely those of the authors and do not necessarily represent those of their affiliated organizations, or those of the publisher, the editors and the reviewers. Any product that may be evaluated in this article, or claim that may be made by its manufacturer, is not guaranteed or endorsed by the publisher.
